# Metabolic Profiling Analysis of Liver in Landes Geese During the Formation of Fatty Liver *via* GC-TOF/MS

**DOI:** 10.3389/fphys.2021.783498

**Published:** 2022-01-03

**Authors:** Yuzhu Yu, Wentao Lyu, Zixian Fu, Qian Fan, Yingping Xiao, Ying Ren, Hua Yang

**Affiliations:** ^1^Hubei Key Laboratory of Animal Nutrition and Feed Science, Wuhan Polytechnic University, Wuhan, China; ^2^State Key Laboratory for Managing Biotic and Chemical Threats to the Quality and Safety of Agro-products, Institute of Agro-products Safety and Nutrition, Zhejiang Academy of Agricultural Sciences, Hangzhou, China

**Keywords:** metabolomics, fatty liver, fatty acid, overfeeding, Landes geese

## Abstract

Fatty liver production results from the process of overfeeding geese, inducing a dramatic increase in *de novo* liver lipogenesis. To investigate the alteration of liver metabolites by overfeeding, especially lipid metabolites, and the potential pathways causing these changes, 60 Landes geese at 65 days old were raised in three groups with 20 geese per group, namely, the D0 group (free from gavage), D7 group (overfeeding for 7 days), and D25 group (overfeeding for 25 days). At 90 days old, segments of liver tissue were collected from 10 geese of each group for gas chromatography time-of-flight/mass spectrometry (GC-TOF/MS) analysis. A large number of endogenous molecules in the livers of geese were altered dramatically by overfeeding. In the livers of overfed geese, the level of oleic acid was observed to continuously increase, while the levels of phenylalanine, methyl phosphate, sulfuric acid, and 3-hydroxybenzaldehyde were decreased. The most significantly different metabolites were enriched in amino acid, lipid, and nucleotide metabolism pathways. The present study further supports the idea that Landes geese efficiently produce fatty liver, and potential biomarkers and disturbed metabolic pathways during the process of forming fatty liver were identified. In conclusion, this study might provide some insights into the underlying mechanisms of fatty liver formation.

## Introduction

It has been documented that Landes geese have a strong ability to store fat in the liver (Tang et al., 2018). After Landes geese have been overfed for approximately 20 days, their fat level can reach nearly 60% of the goose liver mass (Hermier et al., 1994). Above all, no obvious injury was found in the fatty liver of geese. When overfed geese were fed a conventional diet for 20 days, their liver was restored to the original state. This suggests that there are some protective mechanisms used to protect the liver in the process of goose fatty liver (Davail et al., 2000; Guy et al., 2012), and some kind of metabolic mechanism exists to regulate lipid metabolism for recovery when fatty liver occurs in Landes geese. Therefore, Landes geese should be treated as a good fatty liver model, as they can play an important role in exploring the underlying mechanisms of fatty liver further.

With the improved standard of living, obesity and its associated metabolic syndrome have gained ground on a global scale. In particular, hepatopathy, such as fatty liver, has become a major causative factor of chronic liver diseases in American and European countries and wealthy areas of China (Clark, 2006). In recent years, a growing body of research has explored the metabolism of fatty liver using the fatty liver goose model. The existing findings on the fatty livers of geese suggest that most researchers have examined the changes that occur during the formation of fatty liver at the gene level in liver steatosis involved in lipid, cholesterol, and glucose metabolism has been confirmed (Esau et al., 2006; Lu et al., 2015; Zhang et al., 2018). Metabolomics, which developed after genomics and proteomics, is a study method that analyzes tissues, cells, biofluids, organs, or whole organisms to investigate relevant mechanisms (Nicholson and Wilson, 2003; Wu et al., 2018). Zhao et al., by applying metabolomic methods, showed that the formation of fatty liver was accompanied by significant changes in metabolic profiles of the liver, and these changes were related to glucose and fatty acid metabolism, oxidative stress, and inflammation (Zhao et al., 2020). However, in the course of fatty liver formation, the changes in the metabolic profiles of the liver have not been fully elucidated from the perspective of metabolism. Our previous researched the serum metabolism of Landes geese using a metabolic approach investigated the effects of overfeeding. Additionally, continuously elevated levels of pyruvic acid, alanine, beta-glycerophosphoric acid, and proline and reduced lactic acid levels have been demonstrated (Gong et al., 2020).

**Figure 1:**
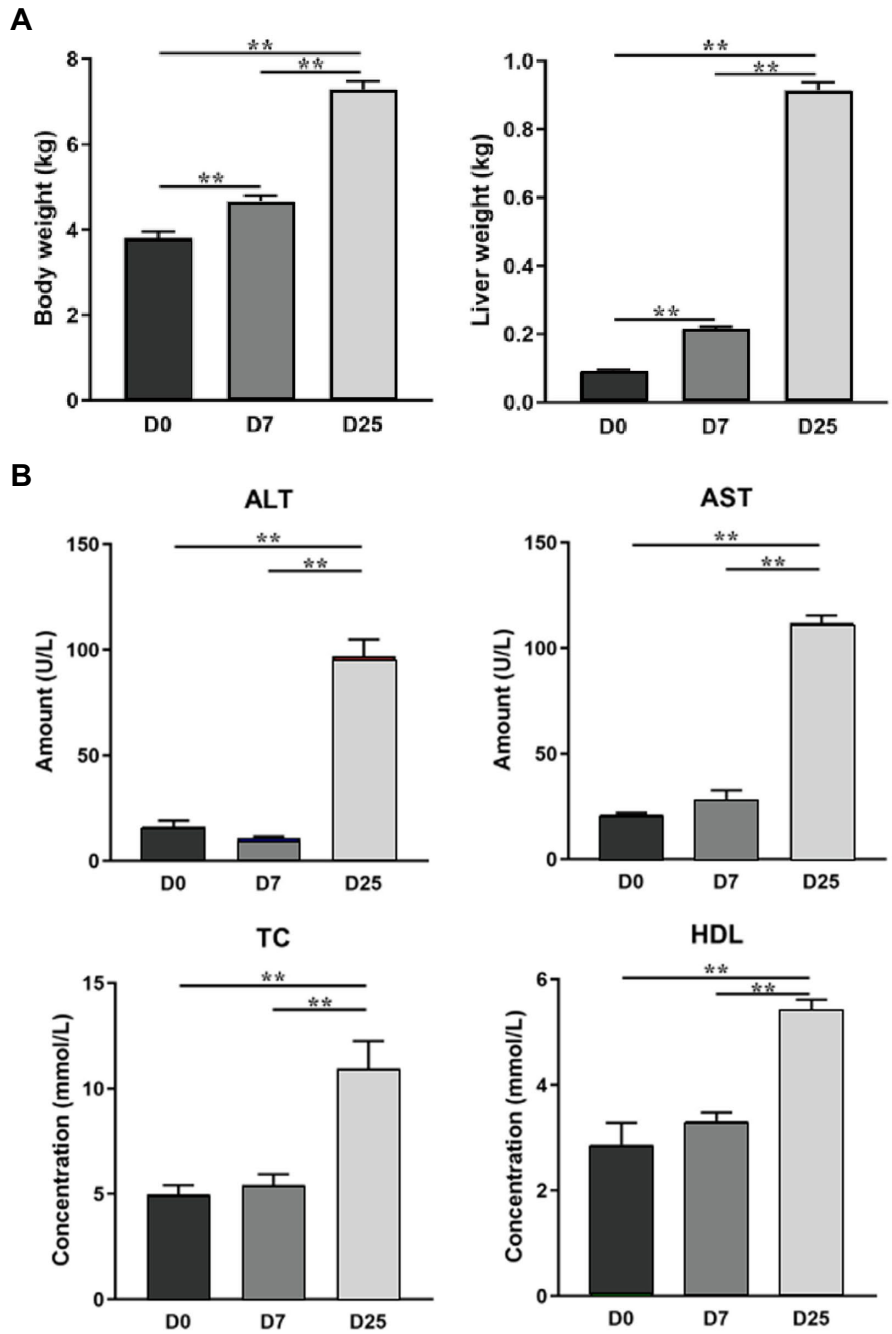
The effects of overfeeding on the body weight and liver weight of Landes geese **(A)** and the serum biochemical parameters of Landes geese **(B)** (Gong et al., 2020). Asterisks (**) represent significant differences with *p* < 0.01.

Previously, we have confirmed overfeeding could produce fatty liver in Landes geese in terms of body weight, liver weight, and serum enzymatic activities. The results showed that the body weight and liver weight significantly increased after overfeeding for 7 and 25 days (Figure 1A) and overfeeding significantly increased (*p* < 0.01) the levels of alanine transaminase (ALT), aspartate transaminase (AST), total cholesterol (TC), and high-density lipoprotein cholesterol (HDL) in the serum of the D25 group (Figure 1B; Gong et al., 2020). In this study, we used the same batch of geese to investigate the changes in the metabolic profile and underlying metabolic mechanism in the liver during the formation of fatty liver in Landes geese. We observed global changes in liver metabolite levels and identified the relevant potential biomarkers and metabolic pathways of Landes geese overfed for different numbers of days with a gas chromatography-time-of-fight mass spectrometry (GC-TOF/MS)-based metabolomics approach and could provide certain data basis for further explore the metabolic changes of liver during fatty liver formation.

## Materials and Methods

The experiment was conducted in accordance with the Chinese Guidelines for Animal Welfare, and the animal protocol was approved by the Animal Care and Use Committee of Zhejiang Academy of Agricultural Sciences (Hangzhou, China). The experiment was conducted at a farm of ChangXing Glory Goose Industry Co., Ltd. (Huzhou, China).

### Experimental Animals and Samples Collection

The test design was based on our previous study (Gong et al., 2020). In brief, sixty healthy male Landes geese of similar weight (65 days old) were randomly assigned to one of three groups (20 geese in each treatment), including one control group and two experimental groups. Treatment included: (1) D0 group: geese fed a regular diet, without any special treatment; (2) D7 group: geese overfeeding on d 19 and lasting for 7 days; (3) D25 group: geese overfeeding on d 1 and lasting for 25 days. The experimental diet was composed of 98% corn, 1% vegetable oil, and 1% salt. The geese were fasted for one night (water was provided) at the end of the overfeeding trial. The next morning, 10 geese of similar weight (90 days old) were chosen from the per groups for liver sampling. The selected geese were slaughtered according to the Administration of Affairs Concerning Experimental Animals of Zhejiang Academy of Agricultural Science, and complete liver stripped from the carcass was weighed. Liver samples were collected and some were stored in −80°C for subsequent analyses.

### Tissues Analysis

The water content and fatty acid composition of liver segments collected from 10 geese per group at 90 days old were measured as previously described (Li et al., 2015; Liu et al., 2020). Liver water content and fatty acid composition in the liver segments were measured separately. Fresh liver samples were used as an analysis of water content by oven drying at 105°C for 24 h to a constant weight. A stored sample of liver was used to determine the fatty acid composition. Briefly, total liver lipids were extracted with petroleum ether/anhydrous diethyl ether (1:1, v/v). Fatty acid methyl esters (FAMEs) were obtained using saponification with a methanolic solution of potassium hydroxide. The organic layer was analyzed using an Agilent 7,890 N gas chromatograph equipped with a flame ionization detector (FID) and a CP-Sil 88 fused silica open-tube capillary column (100 m × 0.25 nm; Agilent Technologies, United States). The settings were: carrier gas: helium; column flow: 40 ml/min; temperature program: 140°C, 140–220°C, 25 min; 220°C, 40 min. The temperatures of the injector and detector were 240°C and 260°C, respectively. The fatty acid peaks were identified by comparing retention times of FAMEs from the sample with the retention time of the standards (Cat#: 18919-AMP; Sigma Chemicals, St, Louis, MO, United States). The amount of fatty acids was normalized to 100%. The extraction and measurement of total lipids refer to the method of Folch et al. (1957).

### Sample Preparation for GC-TOF/MS Analysis

Sample preparation was performed according to the methods of Dunn et al. (2011). A total of 50 ± 1 mg of liver tissue was collected from each sample, placed into 2-ml EP tubes, spiked with 600 μl of extracting solution (methanol and chloroform, 3:1, v/v), and 10 μl of L-2-chlorophenylalanine (1 mg/ml stock in dH2O) and vortexed thoroughly for 30 s. All samples were ground using a grinding mill (Jingxin Industrial Development Co., LTD, Shanghai, China JXFSTPRP-24) for 4 min at 45 Hz and treated for 5 min (incubated in ice water) in an ultrasonic apparatus (Radbon Electronics Co. LTD, Shenzhen, China). After vortexing for 15 s and performing centrifugation at 12,000 rpm for 15 min at 4°C, the supernatant (500 μl) w a s transferred into 1.5-ml EP tubes. Meanwhile, 100 μl of supernatant from each sample was taken as a quality control (QC) sample. Subsequently, the supernatant was desiccated completely in a vacuum freeze drier (Huamei Biochemical Instrument Factory, Taicang, China). The dried samples reacted with 40 μl of methoxy amination hydrochloride (20 mg/ml in pyridine) and incubated for 30 min at 80°C, and 60 μl of bis (trimethylsilyl) trifluoroacetamide reagent (BSTFA, 1% TMCS, v/v; REGIS Technologies, United States) was added to each sample and incubated for 1.5 h at 70°C. When the mixture was cooled to room temperature, 5 μl of a standard mixture of fatty acid methyl esters (FAMEs, C8-C16: 1 mg/ml; C18-C24: 0.5 mg/ml in chloroform) was added to the sample. Afterward, all samples were analyzed by a gas chromatography system coupled with Pegasus HT time-of-flight mass spectrometry (GC-TOF/MS; Dunn et al., 2011). Supplementary Table S1 lists and provides references for derivation reagents used in this study.

### GC-TOF/MS Analysis

Analysis refers to the methods of Yang et al. (2017) and Wen et al. (2018). GC-TOF/MS analysis of the extracted samples was performed using an Agilent 7,890 gas chromatograph (Agilent Corporation, Santa Clara, CA, United States) system coupled with a Pegasus HT time-of-flight mass spectrometry (GC-TOF/MS) system. According to the requirements of the test standard, the system was equipped with a DB-5MS capillary column (30 m × 250 μm × 0.25 μm, J & W Scientific, Folsom, CA, United States) coated with 5% diphenyl cross-linked with 95% dimethylpolysiloxane. In splitless mode with helium as the carrier gas, an aliquot (1 μl) of the sample was injected. Helium was used as the carrier gas, the front inlet purge flow was 3 ml/min, and the column flow was set at 1 ml/min. The initial oven temperature was set to 50°C, held for 1 min, raised to 310°C at a rate of 10°C/min, and maintained for 8 min at 310°C. Set the temperatures of the front injection (280°C), transfer line (280°C), and ion source (250°C), respectively. The energy was −70 eV in electron mode with a solvent delay time of 6.1 min. The mass spectrometry data were collected under the condition of in full-scan mode (m/z range: 50–500; rate: 12.5 spectra per second). Analysis of the QC sample was performed at regular intervals (every ten samples) to monitor the stability of the instrument.

### Statistical Analysis

Raw GC-TOF/MS data were first analyzed using Chroma TOF (version 4.3X, LECO) software for peak extraction, baseline correction, deconvolution, peak alignment, and peak integration. The GC-TOF/MS data were normalized by the area of the internal standards (IS). Relative quantification was performed according to peak area metabolite/peak area internal standard (Dunn et al., 2011). The LECO-Fiehn Rtx5 database was used for qualitative analysis (Kind et al., 2009). Peaks detected in <50% of QC samples or relative standard deviation >30% in QC samples were removed (Dunn et al., 2011). Center scaling (standardization) was performed prior to PCA. The data file was scaled with unit-variance scaling (standardization) before all the variables were subjected to OPLS-DA. Multivariate statistical analysis was performed to analyze the resulting normalization using SIMCA software (version 14.1, MKS Data Analytics Solutions, Umea, Sweden), including principal component analysis (PCA; the data were pre-processed using logarithmically transformed and Ctr scaling) and orthogonal projections to latent structure-discriminate analysis (OPLS-DA; Wiklund et al., 2008). The score plot of PCA shows the systematic clusters among the observations. High-level group separation can be obtained and the variables responsible for classification can be understood by supervised OPLS-DA. We identified the potential metabolites in the process of fatty liver formation combined with S-plot obtained from OPLS-DA analysis. In S-plot diagram, the farther away metabolite ions from origin represent the higher VIP value of the ions, and the higher VIP value represents the greater contribute to the difference between the two sample groups, we selected the ions with VIP >1 and further confirmed to the potential metabolite biomarkers of fatty liver formation. When the variable importance for the projection (VIP) values >1.0 in the OPLS-DA model and *p* < 0.05 in Student’s *t*-test, significantly different metabolites were identified. The heat map of data normalized by Z-score was generated using TBtools. Then, the Kyoto Encyclopedia of Genes and Genomes (KEGG)^1^ was used to search for the related KEGG pathways of the metabolites. The high-quality KEGG metabolic pathway database MetaboAnalyst^2^ was used for pathway analysis and visualization.

The experimental data measured included the water contents of the liver and fatty acid composition. This paper analyzed the data using one-way ANOVA in SPSS 19.0 software. Graphpad Prism 7 software was used for plotting the bar chart and data of fatty acid displayed using logarithmic coordinate axis. Tukey’s multiple comparison test was used to declare significant differences among groups. Significance was set at *p* < 0.05 for all statistical measures. This article displays results in mean ± SEM.

## Results

### Overfeeding Altered the Fat Content and Fatty Acid Composition in the Livers of Landes Geese

**Figure 2:**
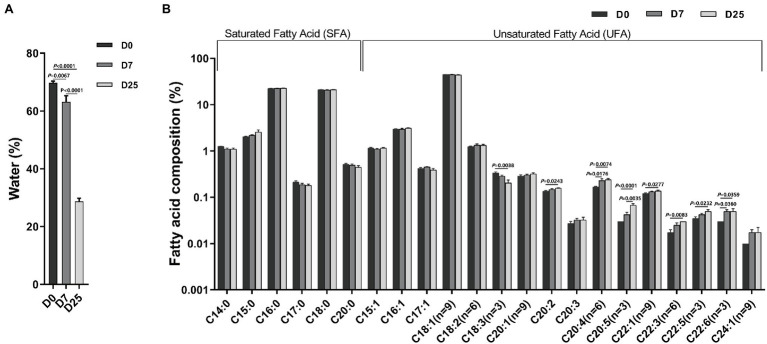
The effects of overfeeding on water content **(A)** and fatty acid composition **(B)** in the livers of Landes geese. Liver samples were collected from geese overfed for 0, 7, and 25 days for the determination of water content and fatty acid composition. Data are expressed as the means ± SEM and analyzed by one-way ANOVA (*n* = 10). The Y axis is a logarithmic coordinate axis. The number at the top of the column is the means. The number on the horizontal line indicate value of *p*. *p* < 0.05 was considered to indicate statistical significance. D0, overfeeding for 0 days; D7, overfeeding for 7 days; D25, overfeeding for 25 days.

To examine the effect of overfeeding on the fatty acid composition in the livers of Landes geese, we determined the water content and fatty acid composition in the liver segments collected from 10 geese per group at 90 days old. As shown in Figure 2A, compared to the D0 group, the contents of moisture in the liver in the D7 and D25 groups were significantly reduced (*p* = 0.0067, *p* < 0.0001). Compared to 7 days of overfeeding, the contents of moisture in the liver were also significantly reduced on the 25th day of overfeeding (*p* < 0.0001). It was obvious that with the increase of the day for overfeeding, the contents of moisture in the liver showed a decreasing trend. To further probe the changes in liver metabolism, we examined the fatty acid composition in the livers of overfed Landes geese on different days. The results are shown in Figure 2B. The effects of overfeeding on total lipids in the liver of Landes geese are summarized in Supplementary Figure S1. The results indicated that the major fatty acids were oleic acid (C18:1n9), palmitic acid (C16:0), and stearic acid (C18:0). C18:1n9 was the most abundant fatty acid, accounting for 44.94 and 45.19% of the total fatty acids in the livers of Landes geese after 7 and 25 days of overfeeding, respectively. When compared with the D0 group, the levels of C20:2 (*p* = 0.0243), C20:4n6 (*p* = 0.0074), C20:5n3 (*p* < 0.0001), C22:1n9 (*p* = 0.0277), C22:3n6 (*p* = 0.0083), C22:5n3 (*p* = 0.0232), and C22:6n3 (*p* = 0.0359) increased in the livers of the D25 groups. In contrast, the level of C18:3n3 decreased (*p* = 0.004). The level of C20:4n6 (*p* = 0.0176) and C22:6n3 (*p* = 0.0360) were significantly increased after 7 days of overfeeding. When compared with the D7 group, C20:5n3 (*p* = 0.0035) levels were significantly higher in the D25 group. The difference was not significant among other groups.

### Metabolite Detection and Identification

The analysis of liver metabolism using GC-TOF/MS yielded 508 peaks after referring to the LECO-Fiehn Rtx5 database. At the same time, NIST library was used for identification. With one peak per compound. Data were p retreated with Chroma TOF 4.3X software to correct the data for missing values, eliminate noise, and normalizes the raw data signals was carried out by internal standard (IS) method, and then 422 peaks were preserved. Among these peaks, 198 compounds were relatively quantified, 224 uncharacterized peak were tagged with “unknown.” The D0, D7, and D25 groups of the total ion chromatograms (TIC) of goose liver samples are shown in Supplementary Figure S2A. Total ion current (TIC) plots of 5 QC samples were shown in Supplementary Figure S2B. The TIC peak retention time and peak area of QC samples overlapped well, indicating that it had good repeatability and instrumental stability for the metabolite detections.

### Metabolic Profiles of GC-TOF/MS Analysis

**Figure 3:**
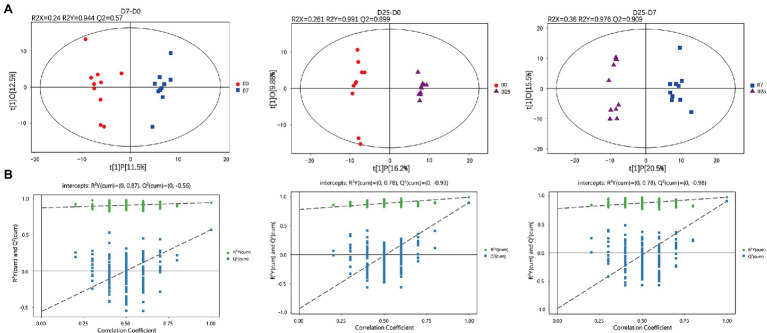
GC-TOF/MS analysis of the livers in Landes geese overfed for 0, 7, and 25 days. Liver samples were collected from geese overfed for 0, 7, and 25 days for GC-TOF/MS analysis. The OPLS-DA score plots **(A)** and OPLS-DA corresponding validation plots **(B)** were generated among the D0, D7, and D25 groups. GC-TOF/MS, gas chromatography-time-of-flight mass spectrometry; OPLS-DA, orthogonal partial least squares discriminant analysis.

To further explore the slight differences in metabolic profiles among all groups, we adopted multivariate statistical analysis methods to analyze metabolomics data. The PCA score plots showed the overall changes in metabolics under the effects of overfeeding. In the result of pairwise comparison (D7 vs. D0, D25 vs. D0 and D25 vs. D7), the values of R^2^X were 0.546, 0.515, and 0.504, respectively. The classification parameters of the three groups were R^2^X = 0.504. They are both >0.4. The results indicated that the D0, D7 and D25 groups showed a clear separation (Supplementary Figure S3). The supervised method, OPLS-DA, was used to screen the variables responsible for differences among these three groups. The OPLS-DA model displayed a clear separation and discrimination among groups (Figure 3A), which indicated that the metabolite profiles in the livers of geese apparently changed. The OPLS-DA model was tested by further permutation tests. The results showed that the, respective, R2Y and Q2 intercept values w e r e 0.944 and 0.57 in the model of the control group and D7 groups; 0.991 and 0.899 in the model of the D0 and D25 groups; and 0.976 and 0.909 in the model of the D7 and D25 groups (Figure 3B). The Q^2^ intercept values were −0.56, −0.93 and −0.98, respectively. And all of the R^2^Y values were close to 1 in our tests, proving that OPLS-DA was established successfully. We identified the potential metabolites in the process of fatty liver formation combined with S-plot obtained from OPLS-DA analysis. In S-plot diagram the farther away metabolite ions from origin represent the higher VIP value of the ions, and the higher VIP value represent the greater contribute to the difference between the two sample groups, we selected the ions with VIP >1 and further confirmed to the potential metabolite biomarkers of fatty liver formation (Supplementary Figure S4).

### Identification of Significantly Different Metabolites in Goose Liver Samples

**Figure 4:**
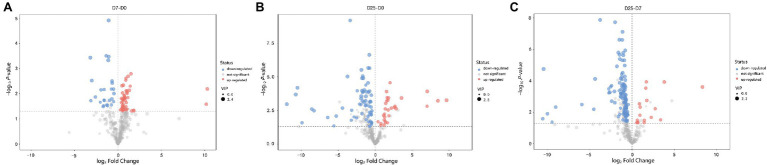
Volcano plots of liver tissue sample metabolites. **(A)** Shows the D7 group compared to the D0 group. **(B)** Shows the D25 group compared to the D0 group. **(C)** Shows the D25 group compared to the D7 group. The red dots represent metabolites that are upregulated, the blue dots represent metabolites that are downregulated, and the gray dots represent metabolites that do not change significantly. D0, overfeeding for 0 days; D7, overfeeding for 7 days; D25, overfeeding for 25 days.

**Figure 5:**
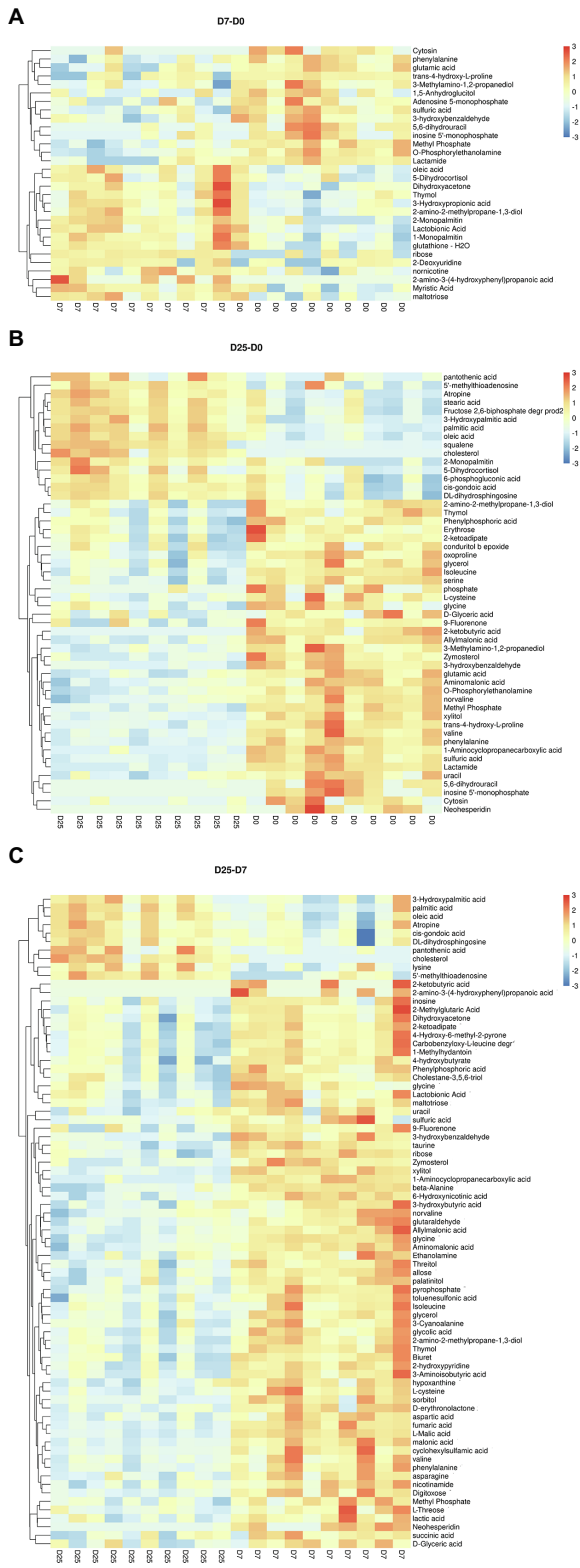
Hierarchically clustered heat map for the significantly different metabolites. **(A)** Shows the D7 group compared to the D0 group. **(B)** Shows the D25 group compared to the D0 group. **(C)** Shows the D25 group compared to the D7 group. The up-regulated and down-regulated metabolites were indicated by different shade colors of red and blue, respectively. D0, overfeeding for 0 days; D7, overfeeding for 7 days; D25, overfeeding for 25 days.

To further probe into the variables in goose livers, the significantly different metabolites were filtered according to the significance test (*p* < 0.05) and VIP value (VIP > 1.0) from the OPLS-DA model. Of the metabolites shown to differ (VIP > 1.0 and *p* < 0.05) between the D7 and D0 groups, 30 were annotated and identified based on the metabolome databases of KEGG and 52 significantly different unidentified peaks (named “unknown”), 52 and 43 between the D25 and D0 groups, and 77 and 53 between the D25 and D7 groups (Supplementary Table S2). There were 95 significantly different metabolites between D25 and D0 (65 down-regulated, 30 up-regulated; Figure 4A), 82 between D7 and D0 (26 down-regulated, 56 up-regulated; Figure 4B), 130 between D25 and D7 (113 down-regulated, 17 up-regulated; Figure 4C). Table 1 shows this result. Subsequently, to further investigate the distinct characteristics of the significantly different metabolites, a hierarchical clustering heat map was plotted (Figure 5). The results from the comparison of the three groups indicated that overfeeding resulted in a continuous increase in oleic acid over time. This was the only significantly different metabolite that was continuously upregulated in the three groups. Although palmitic acid and pantothenic acid were found to have elevated concentrations, there was no difference between the D7 group and the D25 group. There were four metabolites (including palmitic acid, pantothenic acid, cholesterol, and 5′-methylthioadenosine) in the D25 group that exhibited higher concentrations in the D0 group and the D7 group. Meanwhile, overfeeding resulted in a continuous reduced effect in terms of the number of days for some significantly different metabolites. The metabolites included phenylalanine, methyl phosphate, sulfuric acid, and 3-hydroxybenzaldehyde. The other eight metabolites (including glutamic acid, O-phosphorylethanolamine, trans-4-hydroxy-L-proline 2,5,6-dihydrouracil, cytosin, 3-methylamino-1,2-propanediol, inosine 5′-monophosphate, and lactamide) had decreased concentrations after overfeeding for 7 and 25 days. However, compared to the D7 group, the D25 group did not have different concentrations. These 20 metabolites showed a downward trend with the increase in overfeeding days (D25 group vs. D7 group), mostly belonging to organic acids and derivatives (such as 1-aminocyclopropanecarboxylic acid, aminomalonic acid, allylmalonic acid, etc.).

**Table 1:** The number of differential metabolites in the liver after overfeeding.

Item^a^	D7-D0	D25-D0	D25-D7
Increased	56 (16)^b^	30 (15)	17 (10)
Decreased	26 (14)	65 (37)	113 (67)
Total	82 (30)	95 (52)	130 (77)

a *Increased or decreased represents the number of differential metabolites that have a higher or lower concentration in comparison between two groups. Total means the number of total differential metabolites*.

b *The number in parentheses represents the number of metabolites annotated in KEGG*.

### Characterization and Functional Analysis of Key Metabolic Pathways

**Figure 6:**
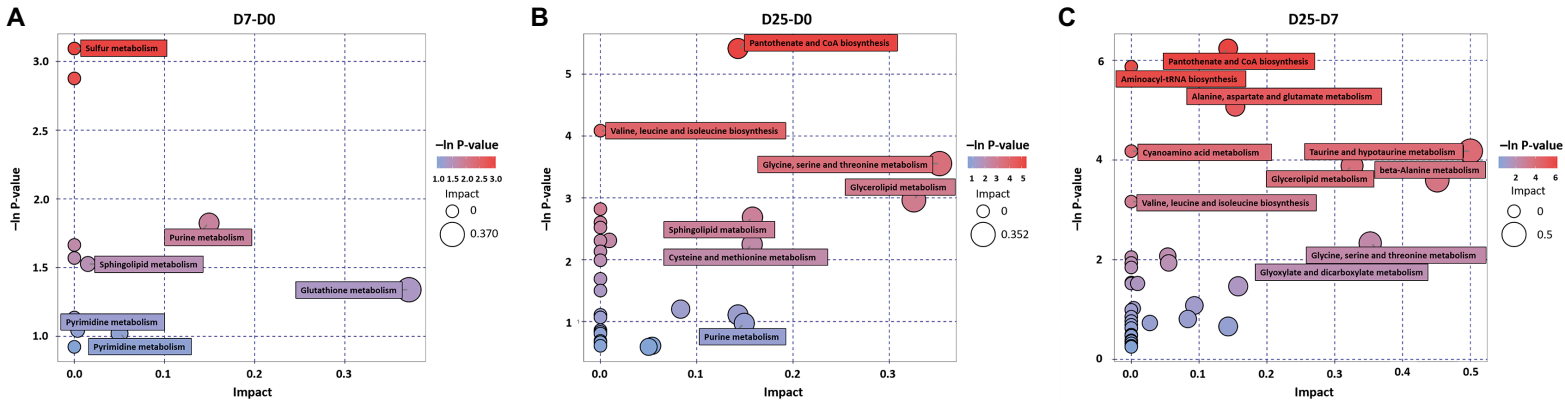
Metabolome view map of significant metabolic pathways. **(A–C)** KEGG pathway enrichment analysis of differential metabolites between the comparison groups (D7 vs. D0, D25 vs. D0/D7). Significantly changed pathways are depicted based on analysis of the enrichment and topology. The bubble size and abscissa jointly indicate the magnitude of the impact factors of the pathway. D0, overfeeding for 0 days; D7, overfeeding for 7 days; D25, overfeeding for 25 days.

To explore the significantly different metabolic pathways in the formation of fatty liver, the significantly different metabolites were imported into KEGG. When we compared the D7 to the D0, the D25 to the D0, and the D25 to the D7, 13, 29, and 38 metabolic pathways were obtained, respectively. They were then filtered based on the -ln value of *p* and pathway impact scores, and 6, 7, and 10 metabolic pathways were enriched, respectively (Figure 6). These key metabolic pathways include various amino acid metabolism (arginine and proline metabolism; glutathione metabolism; valine, leucine, and isoleucine biosynthesis; glycine, serine, and threonine metabolism; cysteine and methionine metabolism; alanine, aspartate, and glutamate metabolism; cyanoamino acid metabolism; taurine and hypotaurine metabolism; beta-alanine metabolism), lipid metabolism (glycerolipid metabolism; sphingolipid metabolism), nucleotide metabolism (purine metabolism; pyrimidine metabolism), pantothenate and CoA biosynthesis, sulfur metabolism, glyoxylate, and dicarboxylate metabolism and aminoacyl-tRNA biosynthesis pathways. Several distinct metabolites (including D-glyceric acid, L-cysteine, valine, glycine, glycine, sulfuric acid, uracil, isoleucine, glycerol, β-alanine, asparagine, aspartic acid, pantothenic acid, O-phosphorylethanolamine, inosine 5′-monophosphate, etc.) were involved in these metabolic pathways, as shown in Table 2.

**Table 2:** Metabolic pathways identified for the significantly different metabolites.

Metabolic pathway	Significantly different metabolites
***D7 group vs. D0 group***	
Sulfur metabolism	(0.457) sulfuric acid^a^ ↓
Purine metabolism	(0.307) inosine 5'-monophosphate ↓
	(0.457) sulfuric acid ↓
Sphingolipid metabolism	(0.477) O-Phosphorylethanolamine ↓
Pyrimidine metabolism	(1.893) 2-Deoxyuridine ↑
Arginine and proline metabolism	(0.501) trans-4-hydroxy-L-proline ↓
Glutathione metabolism	(2.186) glutathione - H2O ↑
***D25 group vs. D0 group***	
Pantothenate and CoA biosynthesis	(3.192) pantothenic acid ↑
	(0.434) valine ↓
	(0.564) uracil ↓
Valine, leucine and isoleucine biosynthesis	(0.579) Isoleucine ↓
	(0.434) valine ↓
Glycine, serine and threonine metabolism	(0.711) D-Glyceric acid ↓
	(0.511) glycine↓
	(0.605) L-cysteine ↓
Glycerolipid metabolism	(0.603) glycerol ↓
	(0.711) D-Glyceric acid ↓
	(2.227) DL-dihydrosphingosine ↑
	(0.363) O-Phosphorylethanolamine ↓
Cysteine and methionine metabolism	(2.471) 5'-methylthioadenosine ↑
	(0.605) L-cysteine ↓
Purine metabolism	(0.003) inosine 5'-monophosphate ↓
	(0.292) sulfuric acid ↓
***D25 group vs. D7 group***	
Pantothenate and CoA biosynthesis	(6.709) pantothenic acid ↑
	(0.615) valine ↓
	(0.250) beta-Alanine ↓
	(0.693) uracil ↓
Aminoacyl-tRNA biosynthesis	(0.531) asparagine ↓
	(0.534) L-cysteine ↓
	(0.467) glycine ↓
	(0.189) aspartic acid ↓
	(1.912) lysine ↑
	(0.662) Isoleucine ↓
Alanine, aspartate and glutamate metabolism	(0.189) aspartic acid ↓
	(0.531) asparagine ↓
	(0.162) fumaric acid ↓
	(0.521) succinic acid ↓
Cyanoamino acid metabolism	(0.549) 3-Cyanoalanine ↓
	(0.467) glycine ↓
Valine, leucine, and isoleucine biosynthesis	(0.662) Isoleucine ↓
	(0.615) valine ↓
Glycerolipid metabolism	(0.594) Dihydroxyacetone ↓
	(0.500) glycerol ↓
	(0.727) D-Glyceric acid ↓
Taurine and hypotaurine metabolism	(0.534) L-cysteine ↓
	(0.289) taurine ↓
beta-Alanine metabolism	(0.250) beta-Alanine ↓
	(0.189) aspartic acid ↓
	(0.693) uracil ↓
Glycine, serine, and threonine metabolism	(0.727) D-Glyceric acid ↓
	(0.467) glycine ↓
	(0.534) L-cysteine ↓
Glyoxylate and dicarboxylate metabolism	(0.411) glycolic acid ↓
	(0.727) D-Glyceric acid ↓

a *The numbers in parentheses indicate the fold change (FC)*.

*↑ and ↓ indicate that the metabolites were upregulated and downregulated in each of the two group comparisons*.

## Discussion

Fatty liver has been considered one of the factors that threatens public health. However, in geese, overfeeding in the liver to form fatty liver is a special way of storing energy (Fournier et al., 1997; Davail et al., 2000). Our previous study found that the BW and liver weights of Landes geese were significantly increased after 7 days and 25 days of continuous overfeeding. On the 7th day and 25th day of overfeeding, the liver weight was notably higher and accounted for 4.57 and 12.56% of the BW, respectively, compared to only 2.40% in the control group (Gong et al., 2020). This suggested overfeeding success in establishing the fatty liver model. On this basis, we analyzed the water and lipid contents and fatty acid composition. The data indicated that the content of moisture in the liver of Landes geese was reduced to 28.73% after 25 days of overfeeding. Similar results were found previously, which showed that the water content in the livers of Landes geese was 35.54% after 19 days of overfeeding (Guy et al., 2012). The liver takes up glucose and converts it into fatty acids, which become incorporated into triglycerides. The main reason for the formation of fatty liver in Landes geese is triglyceride accumulation in the liver (Hermier et al., 1991). This is usually accompanied by structural and content changes in fatty acids of the liver. By comparison, C18:1, particularly C18:1n9, was the most abundant FA in the fatty livers of Landes geese, followed by C16:0 and C18:0. This finding is consistent with recent studies (Tang et al., 2018; Liu et al., 2020). C18:1 is the main FA for the formation of TG, which represented more than 45% of the total FA in the liver after 25 days of overfeeding. These results are in line with those of previous studies on the fatty livers of Landes geese (Hermier et al., 1999; Molee et al., 2005). These data further proved that the model of goose fatty liver was established successfully.

To gain better insight into the metabolic changes that occur in the process of fatty liver formation, we used a GC-TOF/MS method to analyze the liver. The OPLS-DA model indicated that the metabolite profiles in the livers of geese changed noticeably, both after 7 days and 25 days of overfeeding. These results imply that overfeeding significantly changes the metabolic patterns in the liver. Thirty differentially expressed metabolites were identified in the liver after 7 days of overfeeding. These metabolites were involved in a total of 13 metabolic pathways, 6 of which showed significant changes. The number of altered metabolites in the liver was increased by the days of overfeeding. There were 52 common differential metabolites detected in the livers of Landes geese overfed for 25 days, the number of differential metabolite was much higher than that of geese overfed for 7 days. Compared with 7 days of overfeeding, 77 metabolites in the livers of geese overfed for 25 days changed significantly. In summary, the metabolites oleic acid, phenylalanine 1, methyl phosphate, sulfuric acid, and 3-hydroxybenzaldehyde were repeated in three comparisons. For the study of fatty liver formation, they were selected as potential biomarkers, which can be used to determine significant metabolic pathways and can form the basis of therapeutic approaches or diagnostic indicators (Kitano, 2002; Filla and Edwards, 2016).

From the comparison, the liver metabolic changes in the D25 and D0 groups were more similar to those in the D25 and D7 groups. The same metabolic pathways were as follows: valine, leucine, and isoleucine biosynthesis; glycine, serine, and threonine metabolism; glycerolipid metabolism; and pantothenate and CoA biosynthesis. The significantly different metabolites enriched in these metabolic pathways were valine, D-glyceric acid, isoleucine, glycine, glycine, L-cysteine, glycerol, pantothenic acid, uracil, beta-alanine, and dihydroxyacetone. Except for pantothenic acid, these metabolites were all downregulated in the livers of the overfed geese. Among them, D-glyceric acid and valine can be found in two metabolic pathways. After overfeeding geese with high loadings on feed rich in carbohydrates in a short period of time, glucose reached liver cells in abundance, and then through the glycolytic pathway and pentose phosphate pathway, it produced acetyl-coenzyme A (Ac-CoA; Mourot et al., 2000). Ac-CoA is a major integrator of the nutritional status at the crossroads of fat and sugar, and pantothenic acid is a necessary component of CoA, the significant increase in pantothenic acid in the fatty liver suggests that overfeeding exerted an influence on carbohydrate metabolism and gluconeogenesis pathways. It has been reported that pantothenic acid can decrease cell apoptosis and damage by accelerating the biosynthesis of glutathione and effectively controlling lipid peroxide (Ping et al., 2019). Increased pantothenic acid may have a protective effect on liver cells. But how does pantothenic acid increase, we do not know yet. The fatty acids synthesized by geese under the condition of overfeeding far exceeded those degraded by oxidation, causing massive triglyceride accumulation in liver cells (Mourot et al., 2000). Glycerol is the raw material for synthetic triglycerides. The decrease in glycerol in the fatty liver suggests that triglyceride synthesis consumes a large amount of glycerol. D-Glyceric acid was identified as a degradation metabolite of D-serine; it can be converted to carbohydrates and participate in gluconeogenesis to synthesize glucose (Williamson and Ellington, 1975; Abou El-Magd et al., 2010). It is well known that ingestion of a high carbohydrate diet can induce elevated insulin levels, then cause the transcriptional suppression of gluconeogenesis (Lizcano and Alessi, 2002; Manning and Cantley, 2007). In the body, D-serine is largely formed by the transformation of L-serine. L-serine can catalyze the metabolism of fat and fatty acids. The lower level of D-glyceric in the liver could show that more D-glyceric is involved in the regulation of fat metabolism. Moreover, L-serine can be degraded to pyruvate and participates in the TCA cycle, providing the body with a large amount of energy. This is consistent with our previous findings showing that overfeeding led to the intensification of the TCA cycle (Gong et al., 2020). The effects of BCAAs on obesity have been the research focus in recent years (Bianchi et al., 2005; Nilsson et al., 2007; Arakawa et al., 2011). Valine is an amino acid with a branched chain. Prior studies have confirmed that valine is involved in the pantothenate and CoA biosynthesis pathways, which can be converted into pyruvate. This is also a good confirmation of our hypothesis. Bishop et al. showed that adding valine to the diet of mice can increase C18:1n9 and reduce the C18:0 content of the liver (Bishop et al., 2020), and our liver fatty acid results are consistent with this finding. A possible explanation for this might be that the change in valine in the process of fatty liver formation suggests that a weak link may exist between the lipid content of the liver and valine, but further investigation is needed to verify the mechanism.

Liver X Receptor (LXR) can directly or indirectly activate the synthesis of TG in liver by inducing SREBP-1c (Han et al., 2009). Insulin induces SCD-1 expression by increasing nuclear abundance of SREBP-1c or LXRα (Wang et al., 2006). The insulin levels rise in the case of Landes geese being overfed, but our results suggest the biosynthesis of CoA was suppressed. There may be some regulation that down-regulate the expression of SCD-1 by inhibiting the process of increasing LXRα nuclear abundance, inhibit the formation of fat, ultimately. Carbohydrate response element-binding protein (ChREBP), a glucose responsive transcription factor, regulates glycolytic, gluconeogenic, and lipogenic gene expression by encode Lpyruvate kinase (L-PK), glucose 6 phosphatase catalytic subunit (G6PC), FAS, acetyl CoA carboxylase 1 (ACC1), and SCD-1 (Yamashita et al., 2001; Iizuka et al., 2004). There are differences between the ChREBP of mammals and poultry. In mammal, the activity of ChREBP in liver tissue was significantly increased when rats were fed a high carbohydrate diet. ChREBP may be an important factor leading to imbalance of carbohydrate utilization and fat deposition. High carbohydrate diet promotes the expression of the ChREBP gene, which increases the liver’s ability to synthesize TG (Yamashita et al., 2001). In geese, however the ChREBP gene expression was negatively correlated with plasma glucose, insulin and TG in the liver (Hillgartner et al., 1995). According to research findings, the ChREBP had significantly lower expression levels in the hepatocytes with TG overaccumulation (Pan et al., 2010). TG was accumulated massively in liver of overfeeding geese, by limiting synthesis pathways of fatty acid to maintain normal physiological function of liver cells, resulting in a decrease in ChREBP gene expression, ultimately (Dentin et al., 2005). From the structural point of view, contains one nuclear localization signal proline-rich domains, a basic loop-helixleucine-zipper (b/HLH/Zip), and a leucine-zipper-like (Ziplike) domain (Sakiyama et al., 2008). In our research, metabolic pathways of arginine and proline metabolism and valine, leucine and isoleucine biosynthesis are affected, we suspect that this may have to do with the ChREBP gene expression level decreased. But how the works happen are obscure remains to be further investigated.

## Conclusion

Taken together, the above results suggested that the model of fatty liver in Landes geese was successfully constructed by overfeeding for 25 days, which was proven by the changes in fatty acid composition in the liver. Liver metabolites changed dramatically during fatty liver production. The continuously increasing level of oleic acid was observed with reducing levels of phenylalanine, methyl phosphate, sulfuric acid, and 3-hydroxybenzaldehyde in the liver of overfed geese. The most significantly different metabolites were enriched in amino acid, lipid, and nucleotide metabolism pathways. This study provides a detailed understanding of the metabolic mechanism during fatty liver production in Landes geese.

## Data Availability Statement

The original contributions presented in the study are included in the article/Supplementary Material, further inquiries can be directed to the corresponding authors.

## Ethics Statement

The animal study was reviewed and approved by Institutional Animal Care and Use Committee of Zhejiang Academy of Agricultural Sciences (Hangzhou, China). Written informed consent was obtained from the owners for the participation of their animals in this study.

## Author Contributions

YX and HY designed the study. WL and ZF conducted the animal trial and the laboratory work. YY conducted a literature review and analyzed the data. YY and YX wrote and revised the manuscript and approved the final version. YR, QF, and YX provided suggestions on the experimental design and the manuscript. All authors contributed to the article and approved the submitted version.

## Funding

The present study was financially supported by the National Waterfowl Industry Technology System of China (CARS-42-27) and State Key Laboratory for Managing Biotic and Chemical Threats to the Quality and Safety of Agro-products (2010DS700124-ZZ1905).

## Conflict of Interest

The authors declare that the research was conducted in the absence of any commercial or financial relationships that could be construed as a potential conflict of interest.

## Publisher’s Note

All claims expressed in this article are solely those of the authors and do not necessarily represent those of their affiliated organizations, or those of the publisher, the editors and the reviewers. Any product that may be evaluated in this article, or claim that may be made by its manufacturer, is not guaranteed or endorsed by the publisher.

## Footnotes

^1^ http://www.genome.jp/kegg/

^2^ http://www.metaboanalyst.ca/

## Supplementary Material

Supplementary Table S1 | List of reagents. This table lists and provides references for derivation reagents used in this study.

Supplementary Table S2 | The significantly different metabolites in goose liver samples. ^a^The D7 group compared with the D0 group. ^b^The D25 group compared with the D0 group. ^c^The D25 group compared with the D7 group.
