# Supplementary material for: Metabolic Profiling Analysis of Liver in Landes Geese During the Formation of Fatty Liver via GC-TOF/MS
Source: Front Physiol. 2022 Jan 3;12:783498. doi: 10.3389/fphys.2021.783498 (PMC8761942; doi:10.3389/fphys.2021.783498)
Supplement: Supplementary file 3 [file Image_1.pdf]

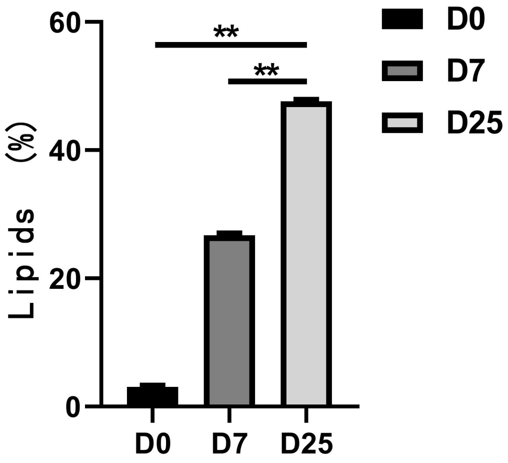

Figure S1 The effects of overfeeding on total lipids in the liver of Landes geese.

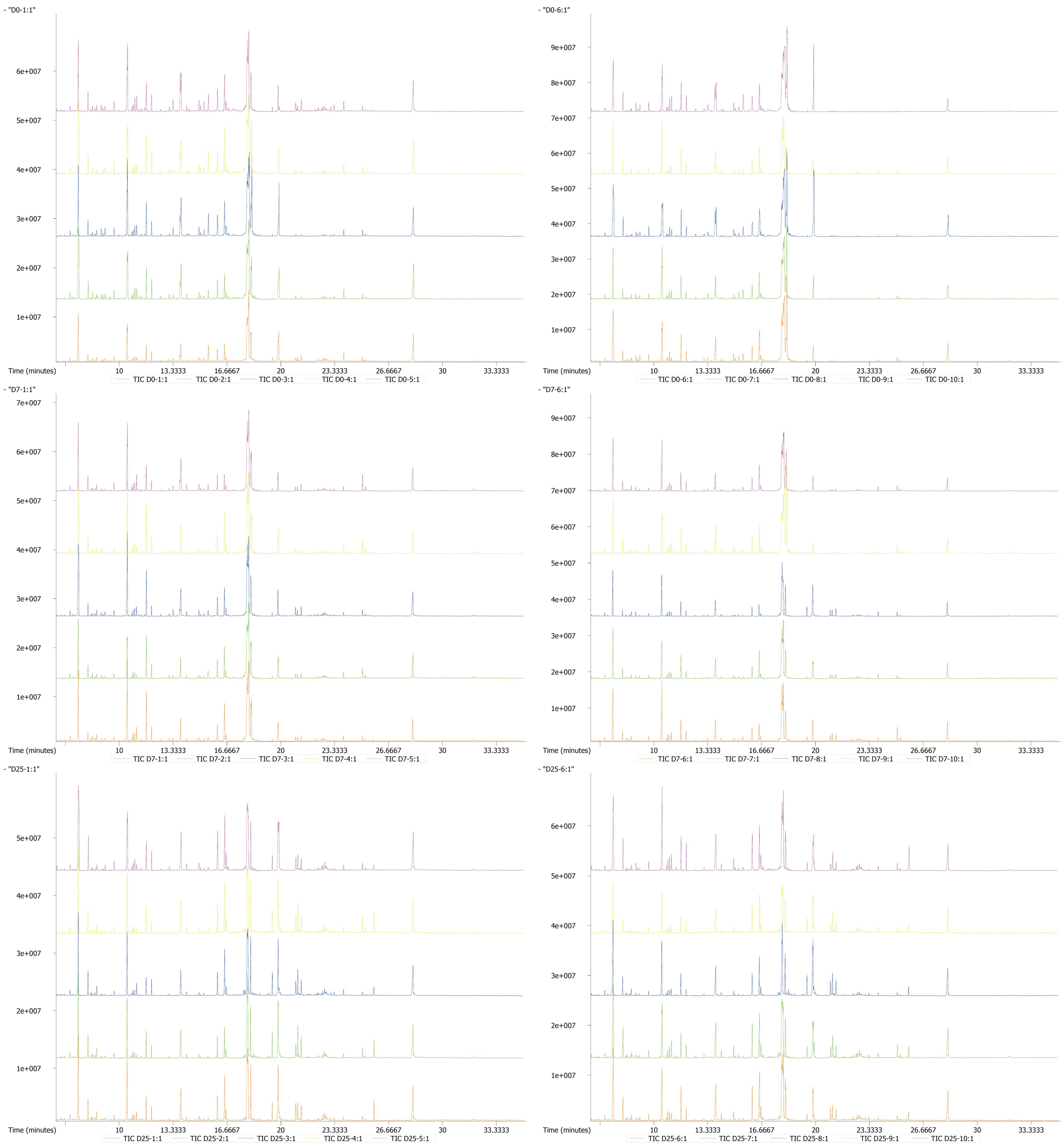

Figure S2A Typical total ion chromatograms (TIC) of goose liver samples from the D0, D7, and D25 groups. The black line represents the D0 group, the red line represents the D7 group and the blue represents the D25 group. D0, overfeeding for 0 days; D7, overfeeding for 7 days; D25, overfeeding for 25 days.

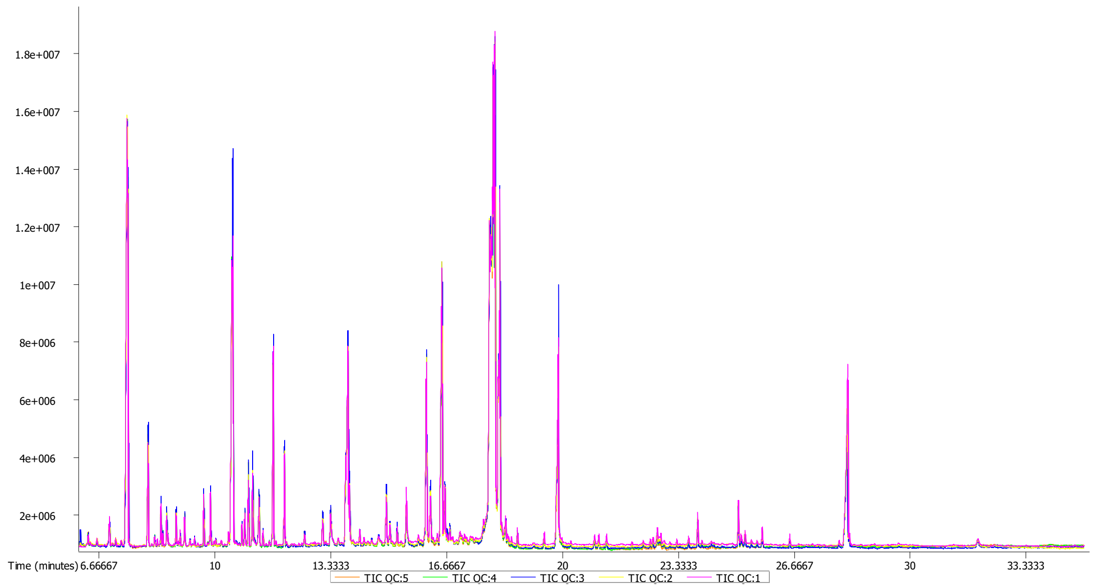

Figure S2B Total ion current (TIC) plots of 5 QC samples.

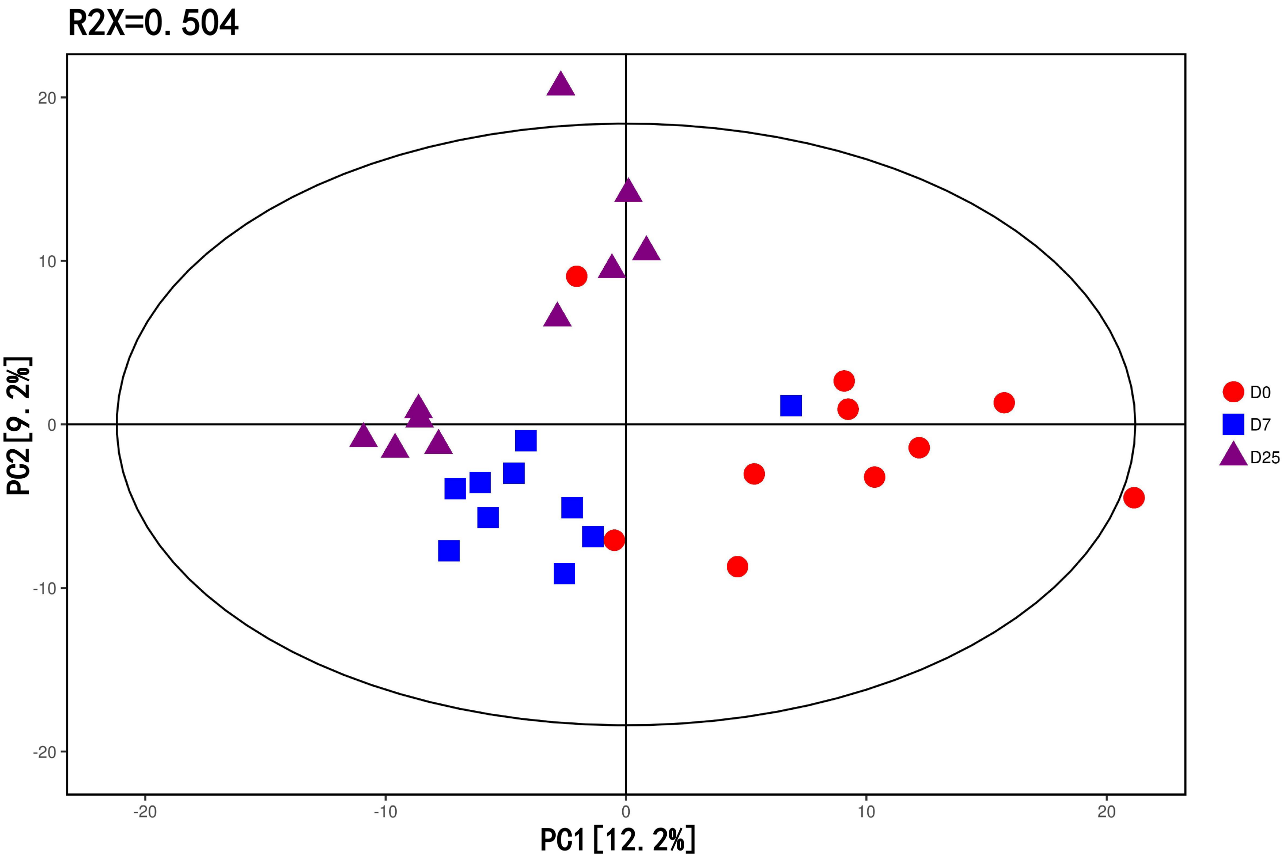

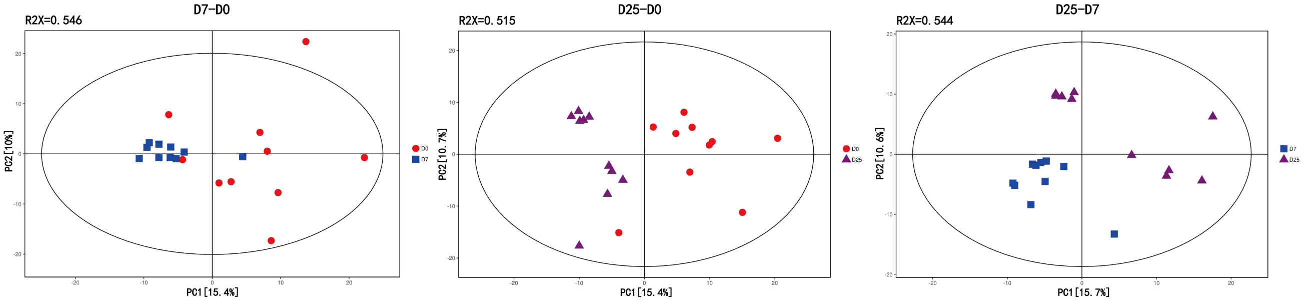

Figure S3 The PCA score plots of samples obtained from the D0, D7, and D25 groups. D0, overfeeding for 0 days; D7, overfeeding for 7 days; D25, overfeeding for 25 days.

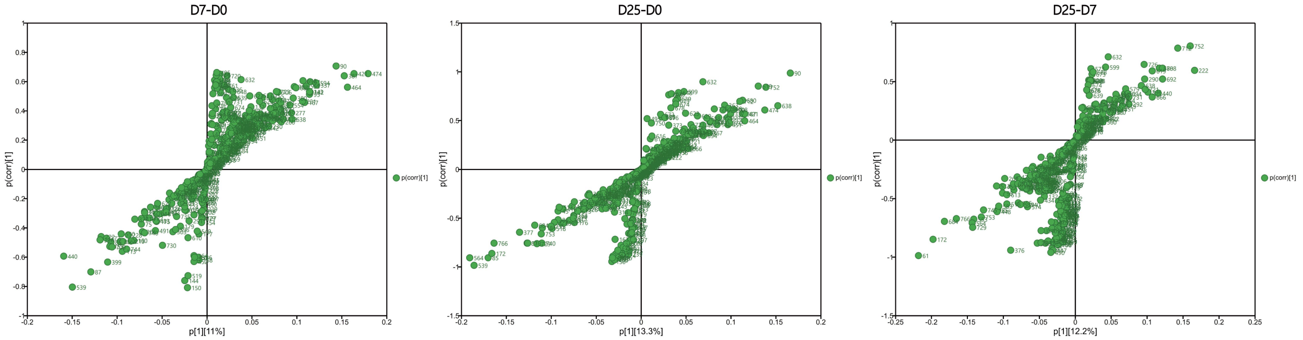

Figure S4 S-plot of OPLS-DA in positive mode. D0, overfeeding for 0 days; D7, overfeeding for 7 days; D25, overfeeding for 25 days.
